# Gray matter density alterations in idiopathic generalized epilepsy patients: a double inversion recovery Magnetic Resonance Imaging study

**DOI:** 10.1186/s42494-025-00239-x

**Published:** 2026-02-03

**Authors:** Yang Cai, Lingyan Mao, Yi Yan, He Wang, Caizhong Chen, Wei Sun, Jing Ding, Xin Wang

**Affiliations:** 1 Department of Neurology, Zhongshan Hospital, Fudan University, 180 Fenglin Road, Shanghai, 200032 China; 2 Department of Radiology, Zhongshan Hospital, Fudan University, 180 Fenglin Road, Shanghai, 200032 China; 3 Institute of Science and Technology for Brain-Inspired Intelligence, Fudan University, Shanghai, 200433 China; 4 CAS Center for Excellence in Brain Science and Intelligence Technology, Shanghai, 200032 China; 5 The State Key Laboratory of Medical Neurobiologyand, MOE Frontiers Center for Brain Science, The Institutes of Brain Science and the Collaborative Innovation Center for Brain Science , Fudan University, Shanghai, 200032 China

**Keywords:** Double inversion recovery, Idiopathic generalized epilepsy, Statistical parametric mapping, Gray matter density

## Abstract

**Background:**

To investigate gray matter abnormalities in idiopathic generalized epilepsy (IGE) patients using double inversion recovery (DIR) sequence combined with statistical parametric mapping (SPM).

**Methods:**

We included a total of 31 IGE patients and 31 healthy controls. All participants underwent 3.0T MRI scans of T1WI, T2WI, FLAIR and DIR sequences. In the IGE group, seizure frequency, severity and electroencephalograph performance were recorded in IGE group. Gray matter intensity was analyzed using statistical parametric mapping through individual and group post-processing procedure of DIR images. Spearman analysis and multiple regression analysis were applied for further analysis of clinical factors and regions exhibiting abnormal gray matter intensity.

**Results:**

The individual SPM analysis of DIR images revealed gray matter abnormalities in 15 out of 31 IGE patients, affecting regions including the temporal lobe, frontal lobe, limbic lobe, occipital lobe, brainstem, insular lobe, parietal lobe, thalamus and cerebellum. Intergroup DIR-SPM analysis comparing IGE patients to healthy controls showed a significant increase in gray matter density in the left temporal lobe (* x* = -44, *y* = -56, *z* = 6, *Z* score = 4.58, *P*_FWE_ = 0.041;* x* = -48, *y* = -40, *z* = -12, *Z* score = 4.54, *P*_FWE_ = 0.047). Generalized spike wave discharges (GSWDs) were positively correlated with the number of voxels exhibiting significantly altered gray matter intensity in each individual with IGE (*P*_FDR_ = 0.032). However, the multiple regression model did not identify any significant brain regions influencing the occurrences of GSWDs.

**Conclusions:**

In the IGE group, various regions exhibited alterations in gray matter density according to either individual or group DIR-SPM analysis. The frequency of GSWDs were correlated with the abnormal voxel count across the entire brain cortex of each individual with IGE.

**Supplementary Information:**

The online version contains supplementary material available at 10.1186/s42494-025-00239-x.

## Background

Epilepsy is a common neurological disorder characterized by abnormal discharges of brain neurons. Idiopathic generalized epilepsy (IGE) represents a prevalent category of generalized epilepsy syndromes, marked by generalized bilateral, synchronous and symmetrical discharges [1]. Idiopathic generalized epilepsies account for approximately 15–20% of epilepsy cases [2]. IGE patients are categorized into four subgroups: childhood absence epilepsy (CAE), juvenile absence epilepsy (JAE), juvenile myoclonic epilepsy (JME), and generalized tonic–clonic seizures (GTCs) alone [3]. These subgroups are distinguished by age of onset, frequency of seizure types, and various combinations of myoclonic and generalized tonic–clonic seizures.

So far, the definition and underlying mechanism of IGE remain controversial. The classification implies that there should be no focal lesions in IGE patients. However, recent studies reported focal gray matter abnormalities and focal semiology features in IGE patients [4–7]. A meta-analysis by Bin et al.[4] showed that IGE patients had increased gray matter volume in the right ventral lateral nucleus and right medial frontal gyrus, while the bilateral pulvinar in had decreased gray matter volume. Additionally, a potential thalamocortical circuit with regional gray matter abnormalities was discovered in IGE patients [5]. Furthermore, a postmortem study demonstrated microdysgenesis and increased neuronal density in the frontal lobe of IGE patients [6], suggesting a potential focal structural etiology. Building on these findings, Seneviratne et al.[7] proposed possible explanations for the presence of focal abnormalities in IGE, including the coexistence of both IGE and focal epilepsy, or the focal onset of generalized seizures in IGE. However, assumptions regarding the focal identities in IGE patients still required further investigation. Understanding the underlying mechanism of focal abnormalities in IGE patients may offer new insights into drug therapy or even provide potential surgical interventions.

The double inversion recovery (DIR) sequence was initially proposed by Shen et al. [8] in 1993 and first used to demonstrate the cerebral cortex by Redpath in 1994 [9]. The sequence is well-known for its sensitivity in improving the detection of focal cortical lesions through either visual inspection or post-processing methods. By utilizing two inverse pulses to suppress cerebrospinal fluid (CSF) and white matter signals, DIR provides direct visualization of gray matter with high contrast against surrounding brain tissue. The DIR sequence has been widely employed in detecting abnormal signals in various diseases involving cortical lesions, such as malformations of cortical development [10], focal cortical dysplasia [11] and drug-resistant epilepsy [12, 13]. Additionally, DIR has enabled high-contrast depictions of cortical lesions in multiple sclerosis (MS) [14]. Furthermore, DIR, when used with post-processing techniques, has improved the identification of epileptic foci. Using DIR-statistical parametric mapping (SPM), Rugg-Gunn et al. [15] identified abnormal signal intensities in gray or white matter in 15 out of 33 routine MRI-negative focal epilepsy patients, with 10 of them corresponding to the epileptic focus. In 2013, a voxel-based analysis by Morimoto et al. [16] in 15 patients with temporal lobe epilepsy demonstrated that the coherence between high signal intensity in DIR-WM and low metabolic area in PET could predict seizure focus laterality, providing a more reliable imaging reference for the pre-surgical evaluation of epilepsy patients.

However, despite focal cortical abnormalities may exist in IGE patients, previous studies have not reported the application of DIR in this context. Given the higher sensitivity of DIR-SPM in detecting brain gray abnormalities, our study aimed to explore these potential gray matter abnormalities in IGE patients using the DIR sequence combined with SPM analysis.

## Methods

### Subjects

We prospectively recruited 31 IGE patients at the epilepsy clinic of Zhongshan Hospital affiliated with Fudan University between May 2016 and July 2022. All IGE patients were diagnosed according to the criteria of the 2017 International League Against Epilepsy (ILAE) [3, 17]. The inclusion criteria for IGE patients were as follows: (i) aged between18 and 65 years with no gender limitation; (ii) presence of generalized spike-wave discharges (GSWDs) in previous or current electroencephalogram(EEG) reports; (iii) normal appearance on conventional brain MRI (including T1WI, T2WI and FLAIR sequences), with no abnormalities such as gray matter heterotopia, white matter gliosis, subcortical nodules, tumors or other lesions; (iv) absence of other medical problems and neurological diseases, and (v) no history of surgery, trauma, or drug abuse.

Additionally, 31 age- and sex-matched healthy controls (HC) undergoing regular physical check-ups at Zhongshan Hospital were enrolled as normal controls if they met the following inclusion criteria: (i) age between 18 and 65 years with no gender limitation; (ii) normal findings on conventional brain magnetic resonance imaging (MRI) including T1WI, T2WI and FLAIR sequences; (iii) no history of neurological diseases or other medical problems, and (iv) no history of surgery, trauma, drug allergies, significant medication use, or familial hereditary conditions.

All participants provided informed consent, which was approved by the Ethics Committee of Zhongshan Hospital (B2024-181R).

### Clinical evaluation

The clinical data collected included age of onset, disease duration, number of antiseizure medications (ASMs) taken, seizure frequency, type, severity, and disease duration. The National Hospital Seizure Severity Scale (NHS3) and VA Seizure type and frequency rating (VA-2) score were also calculated [18].

During the long-term video-EEG monitoring, a 30-min segment of NREM I or II sleep EEG was selected, and the total number of interictal epileptiform discharges in this period was counted for subsequent statistical analysis. Twenty-three IGE patients underwent this EEG examination, and their GSWDs data were further analyzed to investigate the correlation between the number of GSWDs and the abnormal voxel count determined by DIR-SPM.

### MRI protocol

The MRI scan was conducted using a Generic Electric Magnetic Resonance 3 T Sigma HDX scanner with a standard eight-channel phased array head coil. Conventional MRI sequences included axial T1-weighted images (slice thickness = 5 mm, slice count = 21, interval distance = 1.5 mm, repetition time [TR] = 9000 ms, echo-time [TE] = 24 ms, number of excitations [NEX] = 1), T2-weighted images (slice thickness = 5 mm, slice count = 21, interval distance = 1.5 mm, TR = 3400 ms, TE = 110 ms, NEX = 1), and FLAIR images (slice thickness = 5 mm, slice count = 21, interval distance = 1.5 mm, TR = 1750 ms, TE = 150 ms, NEX = 1). DIR sequences were acquired with a slice thickness of 4 mm, a slice count of 20, an interval distance of 1.0 mm, TR of 15000 ms, TE of 80 ms, and one NEX. The MRI scans were performed by radiologist Dr. Wei Sun and interpreted by radiologist Dr. Caizhong Chen. All participant images were reviewed and confirmed as negative by at least two radiologists or neurologists.

### Image processing

The DIR data were preprocessed using SPM12 (http://www.fil.ion.ucl.ac.uk/spm) based on MATLAB2014a. The coordinate origin of the DIR images for each participant was set to the anterior commissure point, and the AC-PC (anterior–posterior commissure) line was manually repositioned to be parallel to the sagittal plane. Subsequently, each participant’s data were normalized to a standard tissue probability map (TPM) template in the stereotaxic Talairach space by 12 linear degrees of freedom and a nonlinear warp in SPM12. To address the defect in the first and last slices of the standard DIR images, the parameter for the bounding box was set to −78, −112, −12; 78, 76, 40. The average DIR image, calculated from 31 healthy controls in SPM, was employed as the template to create a brain mask excluding the cerebellum and cranial bone in Slicer (https://www.slicer.org). Subsequently, the probability map of gray matter, white matter and CSF was segmented using default parameters with reference to the aforementioned mask. The gray matter portion of the segmented images was then smoothed using a Gaussian kernel with dimensions of 8*8*8 mm^3^ voxels for further statistical analysis.

The individual cortical variation map for each IGE patient was generated by comparing the individual preprocessed DIR images with those of all HC candidates using univariate statistical tests on DIR signal intensity. The statistical parametric maps were thresholded at *P* < 0.05, corrected for multiple comparisons using family-wise error (FWE) rate. Significant voxels showing increased or decreased intensity in each individual cortical map were recorded in Supplementary Table S1.

For the group DIR-SPM analysis, a two-sample *t*-test comparing the 31 IGE patients with the HC group was conducted. Two contrasts were defined to detect both increases and decreases in DIR signal intensity. The results were corrected for multiple comparisons using FWE correction with a threshold of *P* < 0.05. The significant voxels were displayed in Xjview (http://www.alivelearn.net/xjview/download-link/) [19].

### Statistical analysis

The significance level for both individual and group DIR-SPM analysis was set at *P* < 0.05, with corrections for multiple comparisons using the FWE method. Voxels with significantly altered gray matter density referred to the voxels that showed changes of gray matter intensity in IGE patients compared to the HC group after DIR-SPM image post-processing. After conducting individual DIR-SPM analyses, the number of voxels that exhibited significantly altered gray matter intensity was included into Spearman analysis.

Kolmogorov–Smirnov tests were utilized to assess the normality of the clinical data. Measurement data that did not conform to the normal distribution were presented as median(interquartile range [IQR]), and non-parametric tests were used for the comparison between groups. Enumeration data were expressed as the number of cases and percentages, and the chi-square test was used for the comparison between groups. Spearman analysis was utilized to explore the correlations between the logarithmic value of the number of voxels with significantly altered gray matter intensity and various clinical factors. Additionally, multiple regression analysis was conducted to predict the primary brain regions associated with GSWD bursts in IGE patients. Statistical analyses were performed using SPSS 24.0. *P* < 0.05 was considered to be statistically significant. In the Spearman analysis, the false discovery rate (FDR) correction method was applied, and results with a corrected *P* < 0.05 were considered significant.

## Results

### Participants

Thirty-one IGE patients and thirty-one healthy controls were enrolled in this study. The clinical characteristics of the participants are summarized in Table 1. The HC group did not differ from the IGE patients in terms of mean age or gender distribution. In the IGE group, the average age of onset was 13.00 (3.25, 17.50) years, and the average disease duration was 13.00 (3.50, 19.00) years. Based on clinical manifestations, three patients were diagnosed with JME, whereas the remaining twenty-eight patients were diagnosed with generalized tonic–clonic seizure alone. Nine IGE patients were on polytherapy (eight patients with 2 ASMs, one patient with 3 ASMs), while the other twenty-two IGE patients received monotherapy. The mean NHS3 and VA-2 scores for the IGE group were 9.00 (6.50, 10.50) and 22.40 (7.80, 70.00), respectively. In addition, six IGE patients remained refractory to medication despite receiving appropriate pharmacological therapy.

**Table 1:** The demographic and clinical information of the idiopathic generalized epilepsy patients and the healthy controls

	IGE (*N* = 31)	HC (*N* = 31)	*P*-value
Age (years, median [IQR])	24 (19, 26)	24 (22, 27)	0.358^a^
Male/Female (N)	15/16	15/16	1.00^b^
Age of onset (years, median [IQR])	13 (3.25, 17.5)		
Disease duration (years, median [IQR])	13 (3.5, 19)		
Seizure type (N)			
GTCs + myoclonic	3 (9.67%)		
GTCs	28 (90.32%)		
ASMs (N)			
Polytherapy	9 (29.03%)		
Monotherapy	22 (7.09%)		
NHS3 (median [IQR])	9 (6.5, 10.5)		
VA-2 (median [IQR])	22.40 (7.8, 70)		
Drug resistance	6 (19.35%)		

*IGE* Idiopathic generalized epilepsy, *HC* Healthy controls, *IQR* Interquartile range, *SD* Standard deviation, *GTCs* Generalized tonic–clonic seizure, *NHS3* National Hospital Seizure Severity Scale, *VA-2* VA Seizure type and frequency rating score

a Mann–Whitney U test; b χ2 Test

### Individual DIR-SPM analysis

The imaging results of the individual DIR-SPM analysis in the IGE group are detailed in Supplementary Table S1. The individual DIR-SPM analysis results were corrected for FWE at a threshold of *P* < 0.05. Among the thirty-one IGE patients, fifteen patients exhibited altered gray matter intensity in various brain regions, despite having a completely normal appearance on conventional T1WI, T2WI, T2 FLAIR images. The remaining sixteen IGE patients were still negative, showing no significantly altered gray matter intensity by individual DIR-SPM analysis.

**Fig. 1:**
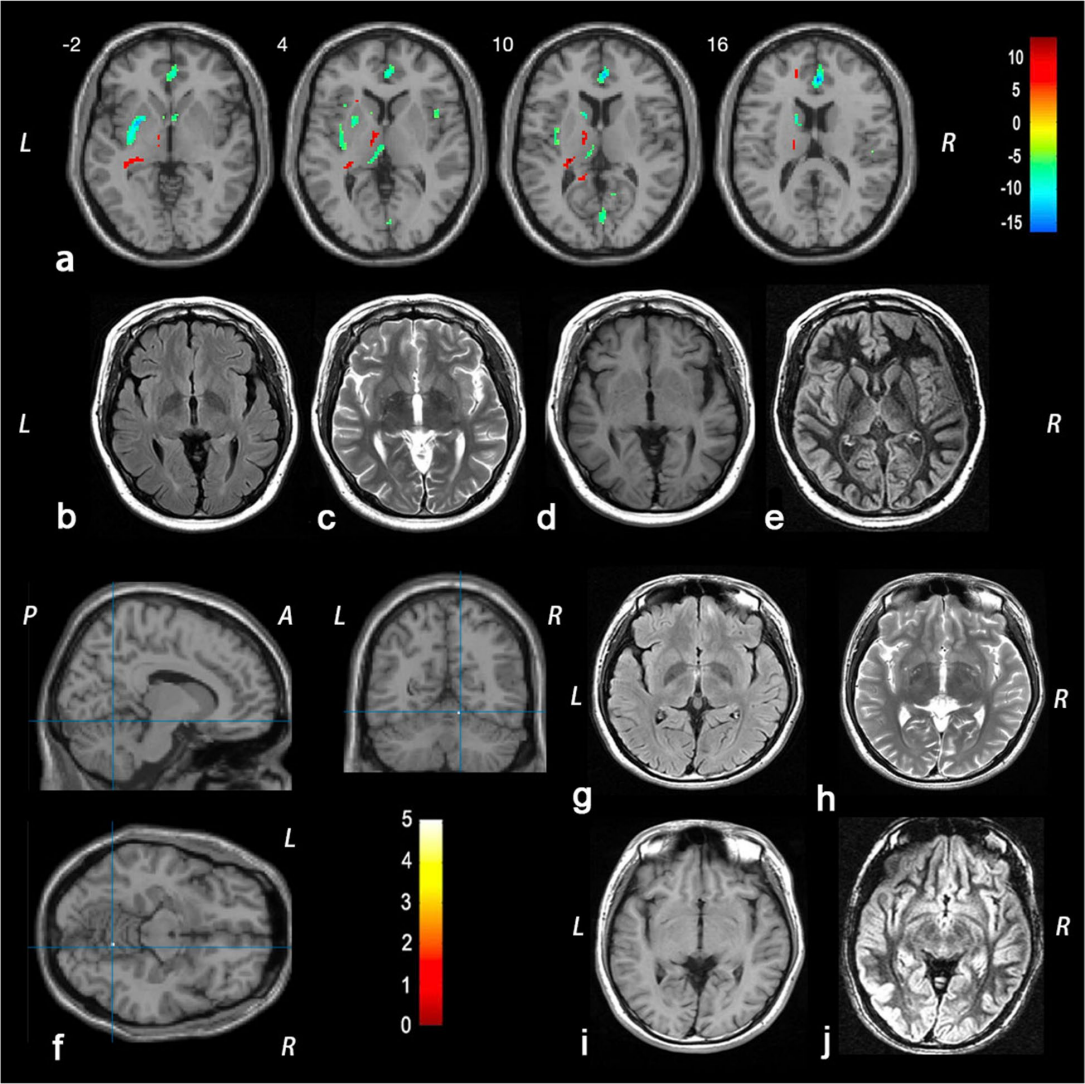
The representative figures of individual analysis results of double inversion recovery- statistical parametric mapping (DIR-SPM). **a** demonstrates a patient (Patient 19) with multiple regions of interests involving a large number of voxels after individual analysis. The individual analysis revealed increased gray matter intensity in the left temporal lobe, bilateral brainstem, left thalamus, left limbic lobe, and left frontal lobe, and decreased gray matter intensity in bilateral limbic lobe, right temporal lobe, bilateral frontal lobe, right occipital lobe, left insular lobe, and bilateral parietal lobe under familywise error (FWE) correction with *P* < 0.05 (**a**). Figure 1f provides an example of an IGE patient (Patient 3) with only one affected brain region (the right cerebellum) (**f**), exhibiting significantly decreased intensity after individual analysis (T = 5.67, *P*_FWE_ < 0.05). However, their conventional MRI imaging, including T1WI (**d**, **i**), T2WI (**c**, **h**), FLAIR (**b**, **g**), as well as the DIR sequence (**e**, **j**), all showed no abnormalities. The color bar represents the T values. L = left; R = Right; A = Anterior; P = Posterior

The mean number of voxels for the fifteen IGE patients with abnormal gray matter intensity was 27, with an interquartile range from 12 to 478. The maximum voxel count reached 1693 in Patient 19 (Fig. 1a-e). The minimum voxel count was only 1 for Patient 3 (Fig. 1 f-j) and Patient 23.

The brain regions exhibiting abnormal gray matter intensity in individual DIR-SPM analysis included the temporal lobe, frontal lobe, limbic lobe, brainstem, occipital lobe, insular lobe, parietal lobe, thalamus and cerebellum. Among the thirty-one IGE patients, 10 out of 31 (32.26%) showed abnormal gray matter intensity in the temporal lobe, while 7 out of 31 (22.58%) exhibited abnormal gray matter intensity in the frontal lobe. The percentages of individuals with abnormal gray matter intensity in the limbic lobe, occipital lobe, brainstem, insula, and parietal lobe among the thirty-one IGE patients were respectively 5/31 (16.13%), 5/31 (16.13%), 4/31 (12.90%), 4/31 (12.90%), and 4/31 (12.90%). Additionally, the ratio for abnormal gray matter intensity in the thalamus and cerebellum was 2 out of 31 (6.45%) and 1 out of 31 (3.22%), respectively.

### Group DIR-SPM analysis

**Fig. 2:**
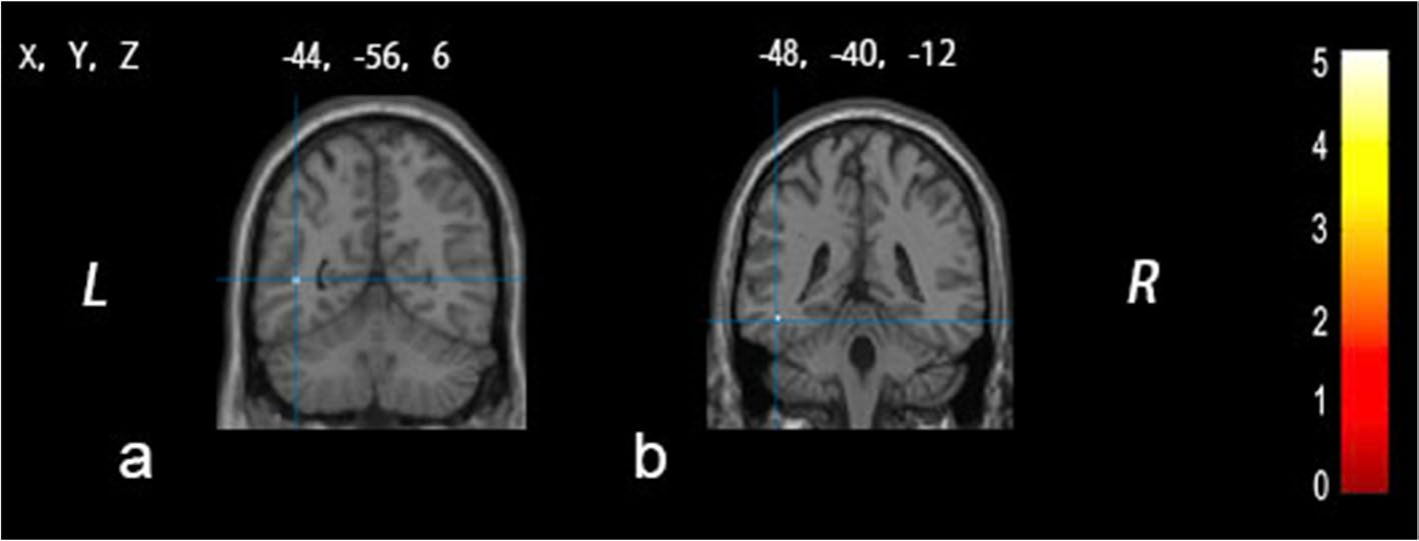
The group analysis results show regions of interests with significant changes in gray matter density in idiopathic generalized epilepsy patients compared to the healthy control group. Figure 2 demonstrates the increased gray matter density (highlighted in yellow) in the left temporal lobe with two clusters (**a**,* x* = −44, *y* = −56, *z* = 6, *Z* score = 4.58, *P*_FWE_ = 0.041; **b**, *x* = −48, *y* = −40, *z* = −12, *Z* score = 4.54,*P*_FWE_ = 0.047), while no decreased gray matter intensity was discovered in other brain regions in idiopathic generalized epilepsy patients compared with healthy controls. The color bar represents the T values

In the IGE group, significantly increased gray matter intensity was observed in the left temporal lobe, with two significant clusters compared to the HC group. The peak MNI coordinates of one cluster was −44, −56,6 (*T* score = 5.05, *Z* score = 4.58, *P*_FWE_ = 0.041, Fig. 2a). The MNI coordinates of the other cluster was −48, −40, −12 (*T* score = 5.01, *Z* score = 4.54,*P*_FWE_ = 0.047, Fig. 2b). No decreased gray matter intensity was found in the IGE group compared with the HC group.

### The relation between the DIR-SPM result and clinical data

**Fig. 3:**
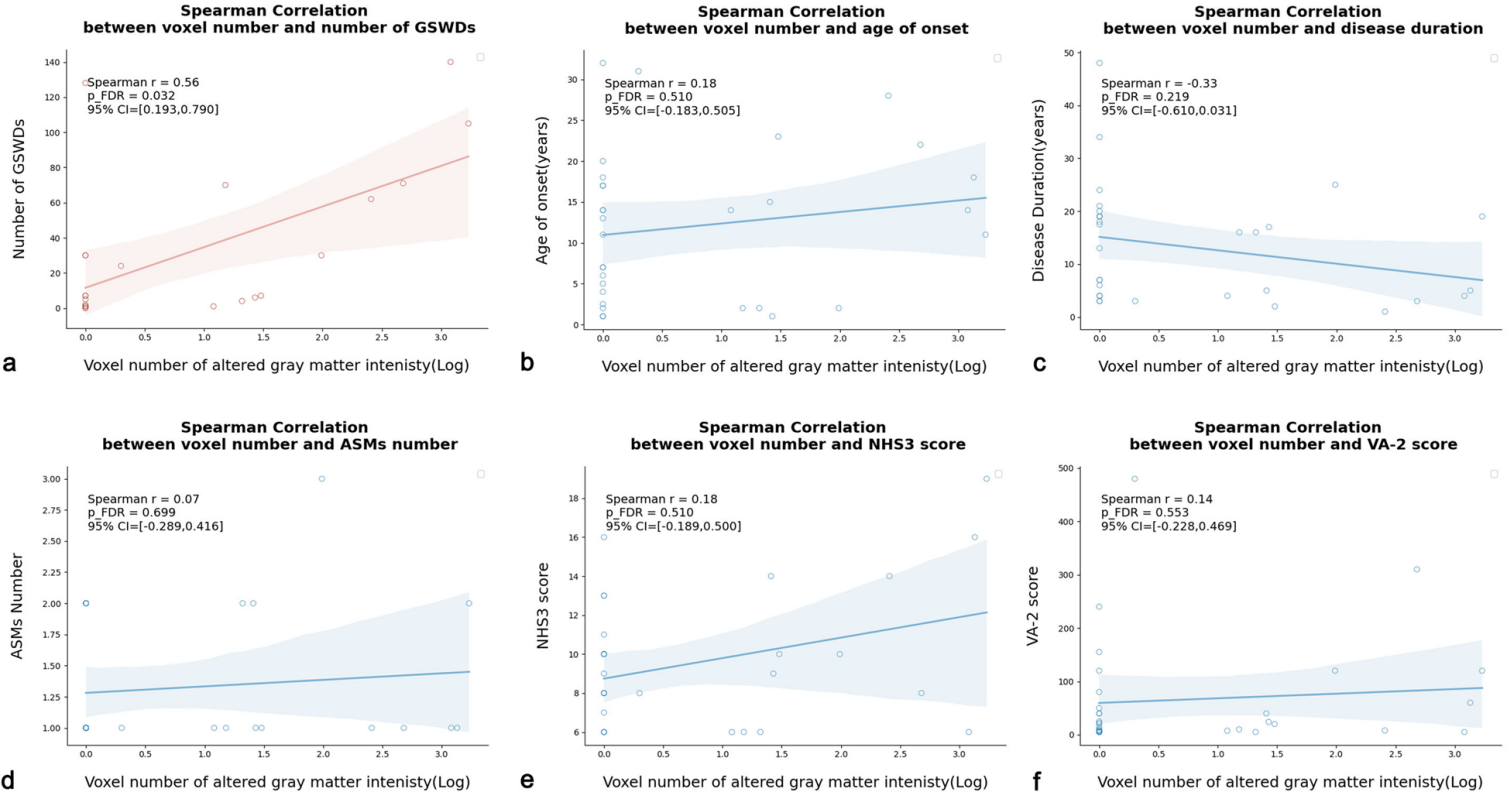
The correlation analysis between the voxel number of altered gray matter density and the clinical factors. Figure 3 depicts a positive correlation between the number of generalized spike-wave discharges and the voxel number of altered gray matter density (**a**, *P*_FDR_ = 0.032). However, under Spearman analysis (*P*_FDR_ > 0.05), no significant correlation was observed between the voxel number and other clinical factors, such as age of onset (**b**), disease duration (**c**), number of antiseizure medications (ASMs) (**d**), NHS3 score (**e**), or VA-2 score (**f**)

The Spearman analysis revealed a positive relationship between the bursts of GSWDs and the number of voxels with significantly altered gray matter intensity (Fig. 3a, *r*_*s*_ = 0.56, *95%CI* = 0.193 ~ 0.790, *P*_*FDR*_ = 0.032). However, the Spearman analysis found no significant relationships between the DIR-SPM voxel number and other clinical factors, including age of onset (*r*_*s*_ = 0.18, *95%CI* = −0.183−0.505, *P*_FDR_ = 0.510), disease duration (*r*_*s*_ = −0.33, *95%CI* = −0.610 ~ 0.031, *P*_FDR_ = 0.219), number of ASMs (*r*_*s*_ = 0.07, *95%CI* = −0.289−0.416, *P*_FDR_ = 0.699), NHS3 score (*r*_*s*_ = 0.18, *95%CI* = −0.189−0.500, *P*_FDR_ = 0.510) or VA-2 score (*r*_*s*_ = 0.14, *95%CI* = −0.228−0.469, *P*_FDR_ = 0.553) (Fig. 3b-f).

Additionally, the average voxel number of altered gray matter intensity was 178.50 (0.00, 696.25) in intractable IGE patients, higher than the value of 1.00(0.00, 18.00) in non-intractable IGE group. However, the Mann–Whitney U test revealed no significant differences on the number of voxels with altered gray matter intensity between intractable and non-intractable IGE groups (*Z* = −1.72, *P* = 0.117).

The multiple regression model was employed to identify the most significant brain regions influencing the number of EEG discharges. The dependent variable (Y) was the number of EEG discharges in each individual, while the independent variables included the temporal lobe (X1), frontal lobe (X2), limbic lobe(X3), brainstem (X4), occipital lobe (X5), insular lobe (X6), parietal lobe (X7), thalamus (X8) and the cerebellum (X9). Each independent variable was assigned a value of 1 or 0 for each individual (1 = Presence of significantly altered gray matter in this brain region according to DIR-SPM; 0 = Absence of significantly altered gray matter in this brain region according to DIR-SPM). The multiple regression analysis indicated that no brain regions were significant predictors of the number of EEG discharges (*F* = 2.685, *P* = 0.064).

## Discussion

To our knowledge, this is the first study to report changes of gray matter density in IGE patients using the DIR-SPM method. Our study utilizing DIR-SPM revealed gray matter abnormalities primarily in the temporal lobe through group analysis. However, individual DIR-SPM analysis showed involvement of various brain regions including the frontal lobe, limbic lobe, occipital lobe, brainstem, insular lobe, parietal lobe, and thalamus. Furthermore, the number of significant voxels exhibited a positive correlation with the GSWDs. However, no specific brain regions were found to be most relevant with the bursts of GSWDs.

Recent studies have demonstrated evidence of focal lesions [4–7] in IGE patients, including focal semiology, focal interictal epileptiform discharges, altered regional gray matter intensity, and focal functional network alterations. The cortical focus theory [20, 21] has become widely recognized to illustrate the focal evidence in IGE patients. The theory holds that the cortical structures provide the excitatory drive, then the thalamus organizes and synchronizes the seizure activity, which forms a reverberating thalamocortical circuitry [22]. While the frontal lobe is often regarded as the primary affected cortex, quantitative electroencephalogram source studies indicate a wider range of extra-frontal areas may be involved, such as the temporoparietal regions [23]. Different studies identified various abnormalities in different brain cortex, making the cortical focus theory unclear. Therefore, further investigation is needed to explore the extent and characteristics of focal cortical involvement in IGE patients.

Double inversion recovery is an MRI sequence specifically designed to highlight gray matter while simultaneously suppressing white matter and CSF. With advancements in MRI parameters [24], DIR has primarily been utilized in diseases related to the cortex, such as focal cortical dysplasia [11], malformations of cortical diseases [10], or MRI-negative focal epilepsy [13]. However, there have been no previous reports on the application of DIR in IGE patients, despite the supposed abnormal structural basis in this population [25]. Therefore, we hypothesized that the DIR sequence, which can provide more detailed cortical information compared to other conventional sequences, would offer greater insights into gray matter abnormalities in IGE patients.

The group DIR-SPM analysis in this study showed that the temporal lobe was the most influenced brain region. Our findings align with previous literature, indicating that the temporal lobe, in addition to the frontal lobe and the thalamus, is also involved in the thalamo-cortical network of IGE patients. For instance, Caplan et al. [26] demonstrated that the volume of bilateral temporal lobe was reduced in 26 CAE patients. Lin et al. [27] observed hippocampal atrophy in JME patients, with the atrophy correlated with memory dysfunctions. A diffusion tensor MRI analysis in JME patients by Kim et al. [23] exhibited white matter alterations in the left temporal lobe. An arterial spin labelling perfusion MRI study by Chen et al. [28] revealed prolonged arterial transit time in the left superior temporal gyrus in IGE patients, indicating cortical dysfunction in the temporal lobe may be related to epileptic activity. Ji et al. [29] found that four corticothalamic networks, including the middle temporal gyrus in the temporal-thalamic network exhibited increased functional connectivity strength in IGE patients. Studies related to electrophysiology also provided evidence of the involvement of the temporal lobe in IGE patients. Tatum et al. [30] reported a focal temporal lobe electroclinical transformation after a typical absence seizure in two absence epilepsy patients. Gelisse et al. [31] observed temporal intermittent rhythmic delta activity in three JAE patients. The animal model of atypical absence seizures also showed that the slow spike-wave discharges related with myoclonic jerks could emanate from the hippocampus, and were associated with cognitive dysfunction [32–34]. Our study found increased gray matter density in the temporal lobe of IGE patients, suggesting possible structural malformations or impaired cortical maturation in the temporal lobe. This may further exacerbate the functional abnormalities in the cortical-thalamic network.

Neuroanatomical and pathological mechanisms may clarify how the temporal lobe is involved in IGE patients and how it relates to the circuity of GSWDs. Previous studies have identified bidirectional fiber connections between the medial thalamic nucleus and the limbic regions, such as the amygdala, CA1 region of the hippocampus and the entorhinal cortex. These connections help explain the regulatory role of the temporal lobe in absence epilepsy [35, 36]. This also clarifies how slow spike and wave discharges, originating from the midline thalamus, can further project to the limbic system during atypical absence seizure [22]. Additionally, direct monosynaptic projections exist from CA1/subiculum of the hippocampus to the medial prefrontal cortex, potentially elucidating the cortico-thalamo-hippocampal circuit in the atypical absence seizure model [22, 37]. Ischemia and hypoxia resulting from GSWDs in IGE, along with secondary toxic effects, may also cause the atrophy of the limbic system, including the hippocampus [38]. Based on the above literature, the changes in gray matter density in IGE can be either a cause of seizure generation and propagation, or the consequence caused by recurrent epileptiform activity.

The gray matter intensity changes in the temporal lobe of IGE patients may be associated with the seizure pattern of GTCs in our study. Zhou et al. [38] reported that volume reduction was found in the hippocampus of IGE patients with GTCs, suggesting that the medial temporal lobe may be more vulnerable in IGE patients with GTCs in IGE patients. Briellmann et al. [39] reported that the degree of the atrophy in the left hippocampus was more severe than that in the right hippocampus after several brief GTCs. The impact of GTCs on the laterality of the temporal lobe by Briellmann et al. [39] is also consistent with the left side suggested in this study. Recurrent GTCs seizure episodes decrease cerebral blood flow, thus increasing the susceptibility of the temporal lobe. Animal experimental studies showed that repeated GTCs could lead to the loss of hippocampal neurons [40]. All IGE patients in our study exhibited the seizure pattern of GTCs, which may explain the increased vulnerability of the temporal lobe in our DIR-SPM group analysis.

The refractoriness of the patients may also influence the structure or function of the temporal lobe in IGE patients. Yeom et al. [41] showed that the failure of initial antiseizure treatment in CAE patients was associated with the involvement of the temporal lobe in the current source. Crespo et al. [42] found that the surface area and local gyrification index was increased in drug-resistant JME patients compared with the drug-responsive group, supporting a neurodevelopmental basis for drug resistance and cognitive impairment in JME. In this study, though no significant differences were found between the intractable and non-intractable IGE patients, there existed a tendency that a wider range of brain regions with abnormal gray matter intensity was compromised in the intractable IGE patients, supporting the results of previous reviews. Therefore, the involvement of the temporal lobe may also stem from drug resistance in some IGE patients included in this study.

Additionally, the individual DIR-SPM results presented in this paper could offer an explanation for the varied conclusions in other IGE imaging studies. Previous research has reported findings of either decreased, increased or unchanged gray matter intensity or volume in various brain regions, including the thalamus, frontal, temporal and parietal gyrus, cingulate cortex, caudate, and putamen of both left and right hemispheres [4, 25, 43–46]. These discrepancies in findings may partly stem from differences in MRI parameters or diverse post-processing MRI software. Moreover, the variability in gray matter intensity among IGE groups may be influenced by the heterogeneity of individual IGE patients, different IGE subtypes, and various clinical influencing factors [47]. The phenomenon is further supported by our individual DIR-SPM results, showing different brain regions and numbers involved in each individual IGE patient.

Nevertheless, most imaging studies of IGE have primarily focused on identifying specific abnormal brain regions, with few exploring the clinical significance of these areas [5, 26, 48–51]. Among the various clinical factors examined, disease duration has been reported to negatively correlate with the volume of the right frontal lobe [48] and thalamic gray matter volume [5, 49]. Other variables such as age, gender, age of onset [26, 48], neuropsychological profiles [50, 51], and antiseizure medications [52] have also been reported to be associated with structural or functional abnormalities in IGE patients. Therefore, in this paper, clinical factors including age of onset, disease duration, number of antiseizure medications, seizure severity score, and bursts of GSWDs were all included in the further analysis.

Our results also revealed a positive correlation between the bursts of GSWDs and the number of voxels with abnormal gray matter intensity. The similar results were also reported in other studies. A correlation study between quantitative EEG and MRI by Betting et al. [47] suggested that subtle gray matter abnormalities were associated with epileptiform discharges observed in the EEG of IGE patients. GSWDs are the EEG hallmark of IGE, and are often accompanied by a decrease in the BOLD signal and reduction in cerebral blood flow in a wide range of brain regions, including the frontal, temporal and the parietal lobe [53, 54]. The GSWDs generated and propagated in the thalamo-cortical circuit can cause abnormalities during the neurodevelopmental process of the cortex [55], abnormal neuronal connections and loss of neuron function[47], as well as the cortical ischemia or hypoxia [53], which in turn lead to cortical changes. EEG/fMRI studies in IGE patients have revealed that during spike wave discharges, there is a notable deactivation of the association cortex. The phenomenon was thought to be a consequence of seizure-induced injury to the cortex, as well as the disruption of its afferent connections during generalized spike wave seizures [56].

In our study, the distribution of GSWDs was further explored using multiple regression analysis. However, no significant brain region was found to correlate with GSWDs. Our findings suggest that GSWDs may be linked to the widespread abnormalities of gray matter instead of a specific brain region. This indicated that a broader range of brain regions rather than a single one was involved with the GSWDs. The cortical focus theory may provide a useful framework for understanding this phenomenon [7]. Usually, this cortical initiation region of GSWDs is the frontal lobe. However, individual differences lead to the fact that the initiation region can include a wider range of other brain cortex beyond the frontal lobe, including the temporal or the parietal lobe [23]. Therefore, the similar individual variability observed in our individual DIR-SPM analysis supports the conclusion that GSWDs are associated with a broader range of brain regions rather than a specific area.

Our study had several limitations. Firstly, the DIR images were acquired using an eight-channel head coil on a 3.0T GE machine. The image resolution in the DIR images was relatively low due to the use of an eight-channel coil. This low resolution may have reduced the sensitivity and specificity of the statistical results obtained after normalization, segmentation and smoothing procedures of the original DIR images. Since the application of DIR postprocessing in our IGE patients was only a pilot study, the parameters of the DIR sequence, particularly with higher channel coils, should be optimized in further studies. Secondly, the size of our study population was relatively small. Furthermore, the number of IGE patients was further reduced in the subsequent clinical analysis, as only twenty-three of them underwent EEG inspections lasting more than two hours in our hospital, which allowed for the calculation of the number of GSWDs. The reliability of our study findings would benefit from further investigation involving larger populations in future research. Lastly, our study is cross-sectional, so its findings cannot directly guide future medication or surgical treatment for IGE patients. The gray matter density changes we observed are likely the consequence of cortical damage caused by recurrent GTCs or GSWDs, rather than the initial structural abnormalities at the disease onset. Therefore, for IGE patients, the medication treatment plans still mainly rely on ASMs for generalized seizures, with the management of intractable IGE patients more focusing on the thalamic stimulation through deep brain stimulation or responsive neurostimulation, rather than the resection of focal cortex in surgery. To clarify the causal relationship, the data of gray matter density at follow-up, or the relationship between EEG source localization of GSWDs and the gray matter density could be further analyzed in the future.

## Conclusions

In summary, gray matter abnormalities varied among individual IGE patients, with different brain regions affected in each case. The group DIR-SPM study revealed that the temporal lobe exhibited the most significant alterations in gray matter intensity within the IGE group. GSWDs in IGE patients were associated with extensive abnormal brain cortex rather than specific brain regions. Confirmation of the focal characteristics of IGE patients would be better achieved through stereo-electroencephalogram (SEEG) or postsurgical pathology in future studies.

## Supplementary Information

Supplementary Material 1.

## Data Availability

The data are available from the corresponding author upon reasonable request.

## Abbreviations

AC-PC: Anterior–posterior commissure
ASMs: Antiseizure medications
CAE: Childhood absence epilepsy
CSF: Cerebrospinal fluid
DIR: Double inversion recovery
EEG: Electroencephalogram
FDR: False discovery rate
FLAIR: Fluid attenuated inversion recovery
FWE: Family wise error
GSWDs: Generalized spike wave discharges
GTCs: Generalized tonic–clonic seizures
HC: Healthy controls
IGE: Idiopathic generalized epilepsy
ILAE: International League Against Epilepsy
IQR: Interquartile range
JAE: Juvenile absence epilepsy
JME: Juvenile myoclonic epilepsy
MRI: Magnetic resonance imaging
NEX: Number of excitations
NHS3: National Hospital Seizure Severity Scale
NREM: Non-rapid eye movement
SD: Standard deviation
SEEG: Stereo-electroencephalogram
SPM: Statistical parametric mapping
T1WI: T1 weighted imaging
T2WI: T2 weighted imaging
TE: Echo-time
TPM: Tissue probability map
TR: Repetition time
VA-2: VA Seizure type and frequency rating
