# Supplementary material for: Gray matter density alterations in idiopathic generalized epilepsy patients: a double inversion recovery Magnetic Resonance Imaging study
Source: Acta Epileptol. 2026 Feb 3;8:4. doi: 10.1186/s42494-025-00239-x (PMC12866400; doi:10.1186/s42494-025-00239-x)
Supplement: Supplementary file 1 — Supplementary Material 1. [file 42494_2025_239_MOESM1_ESM.docx]

Supplementary Table S1. The individual DIR-SPM imaging results in idiopathic generalized epilepsy patients

|  | Number of voxels with abnormality | Z-value | Regions with abnormality by individual DIR-SPM analysis | |
| --- | --- | --- | --- | --- |
|  |  |  | Significantly increased GM intensity | Significantly decreased GM intensity |
| PA 1 | 0 | 5.67 | NA | NA |
| PA2 | 98 | 5.67 | Bil. temp. | L. front. + Bil. temp. |
| PA3 | 1 | 5.67 | R. cerebellum | NA |
| PA4 | 0 | 5.67 | NA | NA |
| PA5 | 0 | 5.67 | NA | NA |
| PA6 | 1201 | 5.66 | Bil. brainstem + Bil. temp. + L. thalamus + L. front. + L. limbic. | Bil. limbic. + Bil. front. + L. temp. +L. occ. + Bil. insula+ L. thalamus + L. par. |
| PA7 | 0 | 5.67 | NA | NA |
| PA8 | 0 | 5.67 | NA | NA |
| PA9 | 0 | 5.67 | NA | NA |
| PA10 | 21 | 5.67 | NA | L. occ. |
| PA11 | 0 | 5.67 | NA | NA |
| PA12 | 15 | 5.67 | L. front. | NA |
| PA13 | 2 | 5.67 | NA | R. temp. |
| PA14 | 0 | 5.67 | NA | NA |
| PA15 | 0 | 5.67 | NA | NA |
| PA16 | 12 | 5.67 | NA | R. temp. + R. occ. |
| PA17 | 478 | 5.65 | L. brainstem | L. temp. + Bil. front. |
| PA18 | 0 | 5.67 | NA | NA |
| PA19 | 1693 | 5.66 | L. temp. + Bil. brainstem + L. thalamus + L. limbic. +L. front. | B. limbic. + R. temp. + B. front.  + R. occ. + L. insula+ Bil. par. |
| PA20 | 0 | 5.67 | NA | NA |
| PA21 | 0 | 5.67 | NA | NA |
| PA22 | 30 | 5.67 | NA | L. occ. |
| PA23 | 1 | 5.67 | NA | R. temp. |
| PA24 | 1351 | 5.64 | R. brainstem + R. front. | Bil. temp. + Bil. front. + R. insula  + R. limbic. + R. par. |
| PA25 | 259 | 5.67 | R. temp. | Bil. temp. + R. insula + R. par. |
| PA26 | 27 | 5.67 | NA | Bil. temp.+ L. limbic. |
| PA27 | 0 | 5.67 | NA | NA |
| PA28 | 0 | 5.67 | NA | NA |
| PA29 | 26 | 5.66 | NA | R. front. + R. limbic. |
| PA30 | 0 | 5.67 | NA | NA |
| PA31 | 0 | 5.67 | NA | NA |

DIR = double inversion recovery; SPM = statistical parametric mapping; GM =gray matter; PA = patient; R.= Right; L.=left; Bil. =Bilateral; front. =frontal lobe; temp.= temporal lobe; par. =parietal lobe; occ. =occipital lobe; limbic. =limbic lobe
